# Dietary Heavy Metal Exposure among Finnish Adults in 2007 and in 2012

**DOI:** 10.3390/ijerph182010581

**Published:** 2021-10-09

**Authors:** Johanna Suomi, Liisa Valsta, Pirkko Tuominen

**Affiliations:** 1 Risk Assessment Unit, Finnish Food Authority, 00790 Helsinki, Finland; pirkko.tuominen@ruokavirasto.fi; 2 Population Health Unit, Finnish Institute for Health and Welfare, 00271 Helsinki, Finland; liisa.valsta@thl.fi

**Keywords:** exposure assessment, dietary exposure, cadmium, lead, arsenic, mercury, methyl mercury, nickel, chemical hazard, foodborne hazard, risk assessment, FinDiet surveys

## Abstract

For the non-smoking and non-occupationally exposed population in Europe, food is the main source of heavy metal exposure. The aim of the study was to estimate the dietary exposure of the Finnish adult population to cadmium, lead, inorganic arsenic, inorganic mercury and methyl mercury as well as nickel using governmental as well as industry data on heavy metal occurrence in foodstuffs and the data from two national food consumption surveys conducted in 2007 and 2012. The sources of heavy metal exposure were estimated for the working-age population (25 to 64 years) and for the elderly (65 to 74 years). Exposure differences between years and between population groups were compared statistically. The mean exposure of women aged 25 to 45 years to cadmium and lead was statistically significantly (*p* < 0.001) higher, and the methyl mercury exposure lower (*p* = 0.001) than that of women aged 46 to 64 years. For nickel and inorganic arsenic the differences were lower but still statistically significant (*p* < 0.05). Between genders, significant difference (*p* < 0.05) was only seen for lead and nickel. Mean cadmium exposure was significantly higher in 2012 than in 2007. For at least 95% of the adult population, the risk of health damage from mercury or nickel exposure is negligible, but the margin of exposure for lead and inorganic arsenic is small and shows a possible risk of cancer or neurotoxic effects.

## 1. Introduction

Heavy metals are harmful to the human body. In addition to the milder consequences, cadmium, lead, inorganic arsenic, mercury, and nickel, which are assessed here, may cause severe health effects, e.g. on the kidney, cardiovascular system, bones, and neurosystem, particularly the more vulnerable developing neurosystem. The influences on a human’s health vary depending on long-term vs. short term exposure and different target groups. In particular, foetuses and young children, whose organs are still developing, are vulnerable to long-term consequences. Many of the heavy metals can cross the placenta into the foetus, and prenatal exposure has been linked to telomere length shortening (arsenic and cadmium, [1]) and lower birth weight (cadmium and lead, [2,3]). Therefore, it is of importance to study the exposure of women at fertile age, as cadmium and lead have extremely long biological half-times, with elimination occurring years after the intake.

In addition to the adverse effects based on which the tolerable intake levels shown in Table 1 have been determined, associations have been found between heavy metal exposure of adults with e.g., infertility [4,5,6] and other disruptive effects on reproduction [7] as well as osteoporosis [8]. Furthermore, studies reviewed in the European Food Safety Authority’s (EFSA) reports [9,10,11,12,13,14] determining tolerable intake levels also show that many of the heavy metals are genotoxic. The mechanism of genotoxicity through oxidative stress, however, is such that a threshold can be expected to exist and, therefore, only inorganic arsenic is considered to be potentially carcinogenic from dietary exposure.

Literature also indicates that gender has an effect on the severity of the health damage, as cadmium retention is generally higher in women than in men and women are also more susceptible to immunotoxic effects of lead, while men are more susceptible to neurotoxic effects of lead and methyl mercury after early-life exposure [15]. In addition, arsenic-induced cancer appears to have gender differences, as men seem to be more affected by skin effects related to arsenic exposure, although more research on the sex differences in all of the effects of heavy metals is needed [15]. Therefore, studying the exposure sources and exposure levels of men and women separately and at different ages is justified.

**Table 1:** Health-based guidance values (HBGV) for dietary intake of the heavy metals, set by the European Food Safety Authority (EFSA) except ref. [16] and ref. [17] used for comparison. HBGV were set separately for inorganic arsenic (iAs), inorganic mercury (iHg) and methyl mercury (MeHg) instead of total amount of metal as for the other compounds. TWI is tolerable weekly intake and TDI the tolerable daily intake; BMDL is the benchmark dose lower confidence limit and its subscript shows the percent change from baseline level. The values are given per kilogram of body weight (kg bw).

Compound	HBGV and Its Type	Health Effect	Ref.
Cd	TWI, 2.5 µg/kg bw/week	Kidney damage	[10]
Cd	0.5 µg/g creatinine in urine ^1^	Osteoporosis related bone break risk	[16]
Pb	BMDL_01_ 0.50 µg/kg bw/d	Developmental neurotoxicity	[11]
Pb	BMDL_10_ 0.63 µg/kg bw/d	Kidney damage	[11]
Pb	BMDL_01_ 1.50 µg/kg bw/d	Cardiovascular effects	[11]
iAs	BMDL_01_ 0.30 to 8.0 µg/kg bw/d	Cancer risk, especially lung cancer	[12]
iAs	BMDL_0.5_ 3.0 µg/kg bw/d	Cancer risk (lung cancer)	[17]
MeHg	TWI 1.3 µg/kg bw/week	Developmental neurotoxicity	[13]
iHg	TWI 4.0 µg/kg bw/week	Kidney damage	[13]
Ni	TDI 13.0 µg/kg bw/d	Developmental toxicity in animals	[18]

^1^ Corresponds to ca. 0.18 µg/kg bw/d according to conversion factor used in [9].

The slow elimination and resulting body burden of cadmium [10] and lead [11] were taken into account by EFSA when setting the health-based guidance values of Table 1.

The European Food Safety Authority has assessed the dietary exposure of European consumers to cadmium (Cd), lead (Pb), inorganic arsenic (iAs), inorganic mercury (iHg), methyl mercury (MeHg) and nickel (Ni) in several publications [13,14,18,19,20,21]. According to these assessments, based on concentration data (mainly governmental monitoring data) from all EU member states and nationally collected consumption data, a part of the adult population is in risk of exceeding the health-based guidance values determined for the different heavy metals (Table 2). However, the concentration data used by EFSA mainly originates from Central European member states, and the levels of heavy metals in foodstuffs vary between areas and countries because the soil, used cultivars and human intervention (e.g., fertilisation, industrial pollution) all affect the heavy metal content of the foods. Therefore, national assessments are needed for national decision making and possible risk management measures such as consumption advice or attempts to reduce the heavy metal levels in foodstuffs.

This study assesses the dietary exposure of Finnish consumers to Cd, Pb, iAs, iHg, MeHg and Ni based on two nationwide food consumption datasets, collected in 2007 [22] and 2012 [23]. The aim of using two food consumption datasets was to compare the heavy metal exposure levels and sources of the exposure between the two years, considering that with the use of the same occurrence data, all exposure differences are caused by changes in consumption habits. The differences in heavy metal intake by gender and age were also studied. The heavy metal exposure of women of fertile age (in this dataset considered to be 25 to 45 years) was compared against the other age groups and men of the same age.

## 2. Materials and Methods

### 2.1. Concentration Data

The occurrence data on cadmium, lead, arsenic and mercury in foodstuffs were partly collected previously and reported by Suomi et al. [24]. These data were supplemented in this project with newer data from the Finnish Food Safety Authority Evira (currently Finnish Food Authority), Finnish Customs Laboratory, anonymised industrial data given by the Finnish Food and Drink Industries’ Federation, as well as literature data. As most of the data were from control samples, either taken by official authorities or by the industry, the results may be skewed and their concentrations may be somewhat higher than the heavy metal levels of random foodstuffs in the market. Literature data were used for foodstuffs for which there were no national data available. The data in total comprised 7090 cadmium, 7000 lead, 2900 inorganic arsenic, 3690 mercury and 2250 nickel results, that is, analysed samples or values from literature. Most of the analyses were performed by either inductively coupled plasma—mass spectrometry (ICP-MS) or atomic absorption spectrometry (AAS), and inorganic arsenic was measured by liquid chromatography with ICP-MS as detector (HPLC-ICP-MS). The sensitivity of the analysis methods varied between sample matrices and data sources (Supplementary material, Table S9). The concentration data were collected over a long period of time: although practically all of the samples had been measured in the 2000s, the part of the data measured between 2010 and 2018 ranged from 43% (cadmium and lead) to 83% (nickel). Thus, data on different foodstuffs may originate from different periods. While the raw data at sample level cannot be shared, due to confidentiality and agreed protocols for their use in national risk assessment, a food subgroup level summary of the final occurrence data is available in Appendix 2 of [25].

Most of the data on arsenic and all of the data on mercury were measured as total arsenic and total mercury. Arsenic speciation results, i.e., amounts of inorganic As compounds (iAs) and of organic As compounds instead of total arsenic, were available for rice. For other foods, the portion of iAs out of total arsenic was estimated to be 2 % in fish and 3.5% in crustaceans and molluscs [26], 100% in water and 70 % in other foods [12]. For mercury, the same assumptions were used as in [13]: in fish, out of the total Hg, 20% is iHg and 100% is MeHg; in crustaceans and molluscs, out of the total Hg, 50% is iHg and 80% is MeHg; in foods other than fish and seafood, all mercury is iHg. The relative contributions are based on literature cited in [13]. With the use of these factors, the sum of iHg and MeHg in fish and other seafood is higher than 100 % of the total mercury, and thus the estimate is conservative.

For cadmium and lead, the governmental or industry data (foodstuffs produced in Finland or analysed by the Customs Laboratory) comprised most of the consumed foodstuffs, and literature data were utilised only for some less used foodstuffs, for which there were no national analysis results available. Mercury data in fish and seafood were mostly from national analyses, except for some imported, mainly Atlantic, species, for which the Norwegian NIFES data were used. Data on mercury concentrations in plant-based foodstuffs, except cereals, were scarce and therefore the estimates for these foods are mainly based on literature. The governmental data on arsenic and nickel concentrations were supplemented by literature data, although to a lesser extent than the mercury data of non-fish food groups.

### 2.2. Food Consumption Data

Two food consumption datasets were used: FinDiet 2007 [22] and FinDiet 2012 [23] dietary surveys. Both were collected with two consecutive 24 h recalls (48 h recall) from people between the ages 25 and 74 years and living in five areas in different parts of Finland; the methodological details are found in the reports. A common limitation in many food consumption datasets is that the data are based on individual’s recall of consumption, which may contain errors. However, the FinDiet surveys were validated to minimize the error of the data collection. The 2007 dataset comprised 1575 people aged 25 to 64 years, out of whom 754 (421 females and 333 males) were 25 to 45 years old (“people in fertile age”), 358 were people aged 46 to 64 years and 463 people aged 65 to 74 years. The 2012 dataset comprised 1295 people aged 25 to 64 years, out of whom 621 (265 males and 356 females) were between the ages 25 and 45 years, 261 were aged 46 to 64 years and 413 people aged 65 to 74 years. No data were available for people older than 74 years.

The food consumption data received were already disaggregated into food ingredient level and aggregated to ingredient classes using the Fineli food composition and recipe databases [27,28] and the in-house Finessi dietary calculation software. Individual data at day level were used in the calculations, and the weight as well as age and gender of the individuals were also known. The individual food consumption data are available according to the data management policies and procedures of the Finnish Institute of Health and Welfare THL. Aggregated FinDiet 2007 and 2012 food consumption data are available at the EFSA Comprehensive Food Consumption Database (www.efsa.europa.eu/en/food-consumption/comprehensive-database, accessed 1 August 2021).

All subjects gave their informed consent for inclusion before they participated in the FINRISK study, for which the FinDiet survey was a part. The study was conducted in accordance with the Declaration of Helsinki, and the protocol was approved by the Coordinating Ethical Committee of the Hospital District of Helsinki and Uusimaa on 14 November 2006 (FINRISK 2007, project identification code 229/E0/06) and on 8 August 2013 (FINRISK 2012, project identification code 162/13/03/00/11).

### 2.3. Exposure Assessment and Statistical Analysis

The exposure assessment was performed probabilistically using MCRA version 8.2 (online program, developed by Wageningen University & Research, Biometris, Wageningen, The Netherlands) [29]. The settings used were the following:

Foods with only non-detect measurements were not included in the analysis. For foods with some non-detect measurements, the exposure was calculated using the middle bound (MB) scenario where the non-detects were replaced by 50% of the limit of reporting (limit of detection LOD or usually limit of quantification LOQ). The MB scenario was considered to best reflect the dietary exposure to ubiquitous elements like heavy metals. The exposure assessment model was BetaBinomialNormal with logarithmic transformation, and 100,000 Monte Carlo simulations were run for each analysis. For uncertainty analysis with the bootstrap method, 10,000 iterations per resampled set and 100 resample cycles (with both concentrations and individuals resampled) were used. 

Statistical comparison was performed on individuals’ daily exposure data using IBM SPSS Statistics v.25 (IBM, Armonk, NY, USA). Each of the study days for each individual in the studied group was included as a separate data point. A two-tailed *t*-test was used, variances were assumed to be different.

## 3. Results

The sources and levels of heavy metal exposure for Finnish adult population are presented so that comparison can be made between working age people and elderly, between exposure based on the food consumption in 2007 and in 2012, and for the exposure levels, also between men and women.

### 3.1. Sources of Heavy Metal Exposure

**Figure 1:**
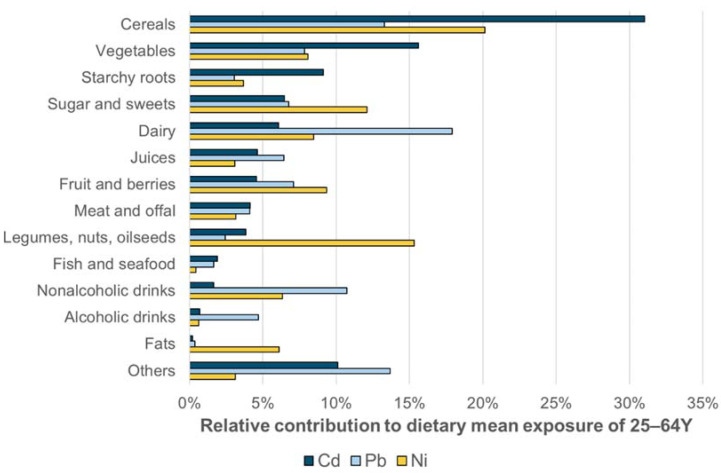
Relative contributions of different food groups to cadmium, lead and nickel exposure of the average consumer of 25 to 64 years, according to FinDiet 2012 consumption data. Food groups are shown in the order of importance for cadmium. The group “Others” in this figure includes eggs, spices and condiments, drinking water, weight loss products (low-cal foods, bars etc.), supplements and combination foods, i.e., dishes that were not divided into ingredient level in the consumption data.

**Figure 2:**
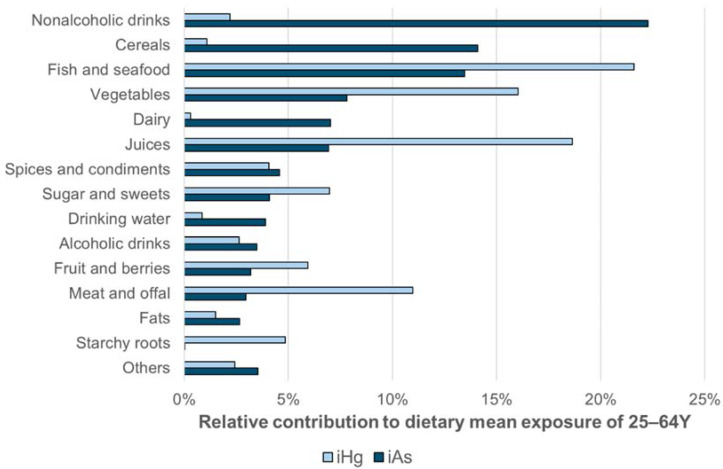
Relative contributions of different food groups to inorganic arsenic and inorganic mercury exposure of the average consumer of 25 to 64 years, according to FinDiet 2012 consumption data. Food groups are shown in order of importance for iAs. The group “Others” in this figure includes legumes, nuts and oilseeds, eggs, weight loss products, supplements and combination foods. The assumptions for the portion of inorganic compounds out of the measured total arsenic or total mercury are detailed under Materials and Methods.

**Figure 3:**
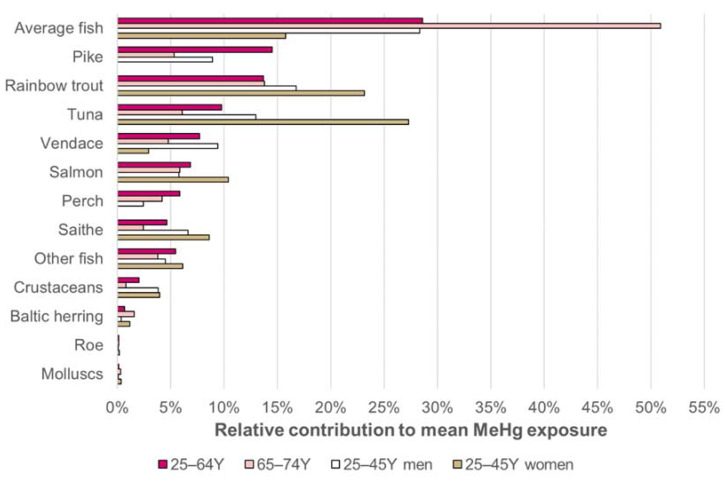
Relative contribution of different fish species and other seafood to mean methyl mercury exposure of different age groups according to the 2012 consumption data. Note that in this data, women of 25 to 45 years did not report consuming pike or perch, possibly due to national recommendations on limiting the use of pike. As seen in Table 3, the mean exposure of the young women was lower than the other groups. “Average fish” was not identified by species and was calculated using the 1:1:1:1 average of mean concentrations of pike, perch, vendace and Baltic herring. These same species had been previously [23] used for nutrition estimates.

Figure 1 presents the relative contributions of different food groups to cadmium, lead and nickel exposure of 25- to 64-year-old Finnish consumers, and Figure 2 presents the same for inorganic arsenic and inorganic mercury exposure. The sources of dietary exposure are shown for the average consumer of this age group according to the FinDiet 2012 consumption data. Figure 3 presents the sources of methyl mercury exposure at age group mean level for several age groups, for the food consumption data collected in 2012. (Supplementary material Tables S1 to S6) compares the relative contributions of the different food groups to total heavy metal exposure for several age groups: general population aged 25 to 64 years, 65 to 74 years, as well as the 25- to 45-year-olds separated by gender. In addition, the results according to the consumption data from 2007 and 2012 are compared in the (supplementary material Tables S1–S6). Table S7 in the supplementary material shows the sources of exposure for the highly exposed (95th percentile) in the group 25 to 64 years according to the 2012 data.

Generally, the foods that are consumed often and in large portions are found among the main sources of exposure. The differences in the occurrence of the studied heavy metal also play a role in determining the main sources. Thus, in Figure 1, dairy is a fairly important source of the three heavy metals based on the high use levels, although the concentrations in dairy products are generally low. On the other hand, nuts and oilseeds are a large source of nickel even though the consumption of these food items in Finland is still low. For the high consumer (95th percentile of exposure distribution), the foods with the highest occurrence of the heavy metal are relatively larger sources of exposure than for the average consumer.

### 3.2. Levels of Heavy Metal Exposure

**Figure 4:**
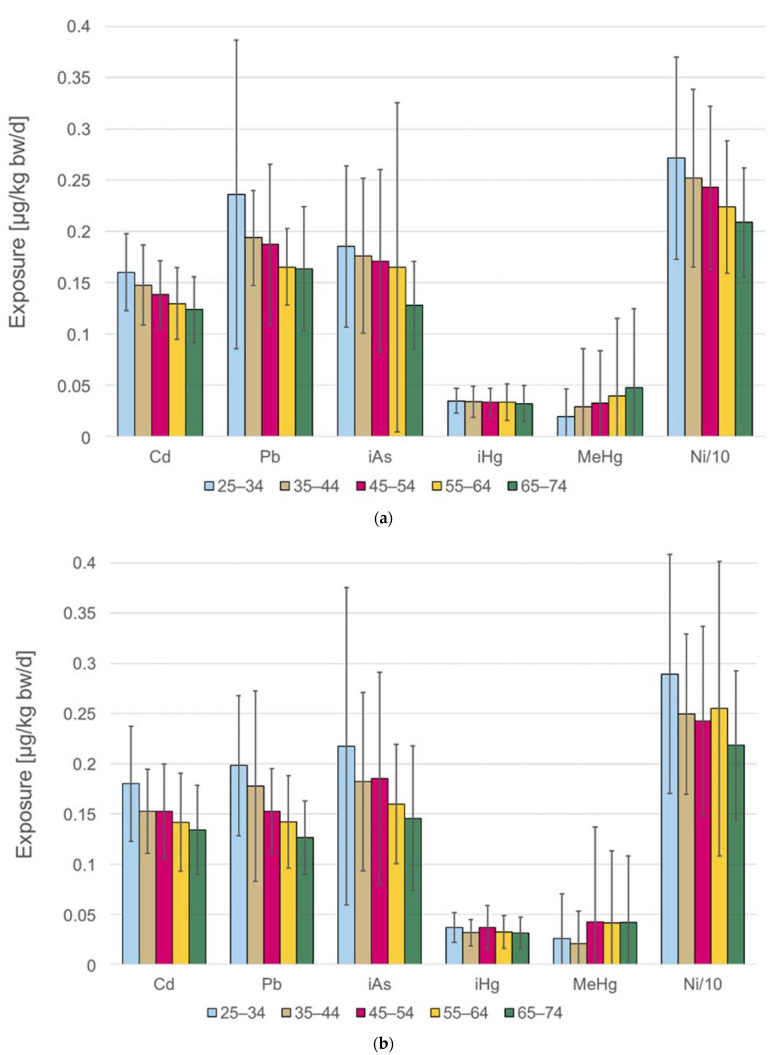
Mean exposure to the heavy metals in the different age groups; standard deviation is marked by whiskers. Nickel exposure is shown as 1/10 of the estimate for scale purposes. Note that the exposure assessment for Pb used different occurrence data for dairy between the two years, due to a decreasing concentration trend, and therefore comparison should be only made within a survey. (**a**) Exposure with FinDiet 2007 consumption data. (**b**) Exposure with FinDiet 2012 consumption data.

The heavy metal exposure is not static: generally, younger adults were found to have higher exposure to heavy metals than the middle-aged or elderly, due to consumption habit differences. However, MeHg exposure increased with age, as older Finns eat more fish and seafood than the younger ones. Fish and seafood were considered to be the only source of MeHg. Figure 4 shows the mean exposure for each 10-year age group in the survey population of 2007 (Figure 4a) and 2012 (Figure 4b). The exposure for nickel is shown as 1/10 of the estimate in these Figures, as the iHg exposure and the Ni exposure have a roughly 100-fold difference as evident in Table 3. Supplementary Table S8 also shows the comparison by gender for the data shown in Figure 4.

Table 4 discusses the risk from dietary exposure to the studied heavy metals based on the exposure assessment for FinDiet 2012 data. Damage to health from exposure to inorganic arsenic, lead and cadmium is possible for a part of the population. Previously, we have assessed that the current lead exposure in Finland corresponds to a burden of disease 570 DALY/year (disability-adjusted life years per year) [30]. Currently available information has not allowed us to estimate the national burden of disease caused by exposure to other heavy metals.

### 3.3. Statistical Comparison of Exposure Levels between Population Groups and between the Two Years

Statistical comparison between women of 25 to 45 years and men of the same age, and between women of 25 to 45 years and women of 46–64 years, was made based on individual exposure assessed with the 2012 consumption data (Table 5). Likewise, comparison was made between the dietary exposures in the lowest and highest weight quartile of the 2012 data (Table 6). The people in the different weight quartiles were not separated by gender, and the lowest quartile included a higher percentage of women than the highest quartile. We used alpha 0.05 to reject the null hypothesis that the mean exposure in the two groups is the same. Between some population groups and some heavy metals, the difference was statistically highly significant (*p* < 0.001). In order to call attention to the statistically highly significant differences among the significant ones, the *p* values are shown for the comparisons also in the text.

The exposure of 25–45Y women to Cd and Pb was statistically highly significant (*p* < 0.001), and exposure to Ni (*p* = 0.008) and iAs (*p* < 0.05) significantly higher than the exposure of 46–64Y women. The MeHg exposure of the younger age group was, however, significantly (*p* < 0.01) lower than in the older group of women. Compared with men of the same age, the women had significantly higher exposure to Ni (*p* = 0.001) and Pb (*p* < 0.05) than the men. In comparison with the weight quartiles, only MeHg exposure was not highly significantly (*p* < 0.001) higher in the lowest quartile.

Table 7 shows statistical comparison of how the estimated average exposure changes between the two years for (1) 25–45 Y adults of both genders and (2) for 25–45 Y women.

In Table 5, Table 6 and Table 7, exposure of each individual on each of the two survey days was used as one data point in the calculations. In independent samples *t*-tests, variance in the compared groups was assumed to be different.

In Table 7, Cd exposure was highly significantly (*p* < 0.001) higher in 2012 than in 2007 when both men and women were studied together, and significantly (*p* = 0.007) higher also when only 25–45Y women were compared. iAs exposure was significantly (*p* < 0.05) higher in 2012 than in 2007 when the whole age group was studied, but no statistically significant difference was seen when comparing only women.

## 4. Discussion

For methyl mercury, the main risk group is women of fertile age. Methyl mercury exposure is expected to only occur from consumption of fish and seafood, and national consumption advice has been directed particularly for this risk group (see [31]). As the methyl mercury exposure of women of fertile age was below the tolerable weekly intake (TWI), it appears that the advice is being followed.

Women of fertile age are an important risk group also to other heavy metals, particularly lead and nickel, the adverse effects of which are linked to the health of the foetus [18]. Their exposure to these heavy metals is significantly or highly significantly higher than that of men of the same age or of older women. However, the FinDiet survey sampling did not include enough pregnant or lactating women (at the time of giving their food recall interviews) to assess their dietary exposure separately. Therefore this background information was not requested with the data. The Finnish system of maternity clinics gives pregnant women information on foodstuffs to avoid at this time, and therefore the exposure of currently pregnant or lactating women, most likely to transmit the heavy metals into the foetus/baby, may be different from the general exposure assessed here. On the other hand, long-time exposure to lead can affect the foetus also later, as lead from the bones may end up in the blood with changes in the bone metabolism during pregnancy [11]. The estimated lead exposure of adults and that of young children [24] were used to estimate the national burden of disease due to dietary lead in [30]. Of the three chemical hazards assessed in that reference, lead had the highest burden of disease. 

As the dietary exposure to heavy metals is measured relative to the body weight, it is to be expected that light people have relatively higher exposure than heavier people. In comparison of the lowest and highest weight quartile, the lowest quartile had significantly higher exposure to all other heavy metals except methyl mercury.

The estimated dietary exposure (Table 3) was lower than previously estimated by EFSA (Table 2), which was to be expected as the Europe-wide occurrence data are more likely to overestimate the concentrations of contaminants in foodstuffs than nationally collected data. The largest percent difference was seen for cadmium and lead, for which the national occurrence data were the most comprehensive. Heavy metals with data supplemented by EFSA averages, and/or assumptions on the relative portion of the inorganic or organic fraction, had less of a difference with the EFSA estimates.

The main limitations of the current study are:

1. The occurrence data, mainly collected for governmental monitoring purposes, are likely to show somewhat higher concentrations than the average levels in all foods available in Finland, as the sampling is directed to potentially risky products;
2. The sensitivity of the laboratory analysis methods is such that a part of the occurrence data is below the limit of quantification (LOQ). Use of the lower bound (<LOQ = 0) and the upper bound (<LOQ = LOQ) estimates will give the lower and upper end of the estimate, and while the middle bound (<LOQ = 0.5 LOQ) used in this study is more realistic than either of them for ubiquitous compounds like heavy metals, it may overestimate or underestimate the importance of some food subgroups;
3. The occurrence data are measured mostly from food ingredients (raw agricultural compounds or their simple derivatives) and so the effect of food preparation on the heavy metal concentrations is not known, unlike in the total diet study, where the analyses are made of food ready for consumption;
4. The consumption data are collected and coded for the purposes of nutritional assessment, which may occasionally mask some details that can be relevant for risk assessment (e.g., lesser used ingredients not yet included in the Fineli food ingredient database are coded as something else) and, therefore, the ingredients in the consumption data and the occurrence data may display minor differences possibly having a small effect on the final result.

### Food Consumption Trends after 2007 and Their Potential Impact on Heavy Metal Exposure

Food consumption has been monitored in Finland in connection to the Population Health Studies (FINRISK and FinHealth Studies) by the so called FinDiet Dietary Surveys since 1982 [23,32,33]. The recent 20-year trend analysis carried out in comparable study areas and food consumption data collection methods [33] covering the FinDiet Surveys between 1997–2017 showed an overall increase in vegetable, fruit and berry as well as pulses, nuts and seeds consumption and a decrease in wheat and rye consumption, but increase in other cereals, e.g., oat, corn and rice consumption both in men and women. Consumption of pulses, nuts and seeds is still at a very low level among both men and women in Finland. Meat consumption has decreased among men during recent years, but it is still clearly higher among men than among women [33,34]. The consumption of fish and seafood has on average not changed during the past 20 years.

The decrease of cereal products especially after the year 2007 could be expected to decrease cadmium and lead exposure. However, part of the decrease of wheat and rye has been compensated by an increase in rice consumption, which would tend to increase As intakes.

Increase in the consumption of oilseeds is likely to increase the heavy metal exposure, unless they substitute in the diet another significant source of exposure. The concentrations of Cd and Ni in oilseeds can be relatively high, but despite that, from the point of view of total health, the use of oilseeds can still be beneficial. 

Out of the three main region-specific dietary patterns in Europe, Finland belongs to the Nordic one. It is characterized by a higher consumption of animal-derived foods as well as processed and sweetened foodstuffs, including non-alcoholic drinks; added fats and dairy products are also common [35]. The dietary trends in the Nordic countries are also similar. In comparison of food consumption data from the end of the 1990s to the early 2010s, it was seen [36] that vegetable consumption increased in all Nordic countries and fruit consumption in all countries except Denmark, although the goal of five servings/day was only reached by about 13% of the Nordic adult population in 2014. An increasing trend on fish intake was also reported [36], although not all individuals eat the minimum recommended amount of fish. The reported wholegrain intake in the period was around 40–60 g/day in the Nordic countries.

Heavy metal concentrations in berries measured in Finland are generally very low, and the same also applies to many vegetables [25]. However, some plant foods can have high concentrations of heavy metals, in particular seaweeds, and increase in the consumption of these particular products would increase the iAs, Cd, Ni and possibly Pb exposure in the population [25]. Therefore, the effect on the changes in the food consumption depends partly on what types of vegetables and other foods are used.

In a pilot risk-benefit study [37] using national monitoring data also utilized in this work, it was estimated that by increasing the use of wholegrains to 232 g/d recommended by the EAT-Lancet Commission, the Cd exposure of Finnish adults would increase from the current level but the benefits of increasing dietary fiber intake would lead to a much larger decrease of burden of disease. The use of oilseeds at 15 g/d, on the other hand, would not in itself fulfill the daily need of dietary fiber, but it would increase the daily fiber intake by 1.3 g, compared with the 2012 consumption. With this addition, the average dietary fiber intake of men in 2012 would be above the limit used by The Institute for Health Metrics and Evaluation at the University of Washington, U.S. (IHME UW) for low fiber intake, 23.5 g/d and that of women would be close to it. Thus, the portion of the population with too low a fiber intake would decrease, but the study did not have access to additional information needed to estimate how large a population that would be, in order to assess the net effect at the whole population level.

## 5. Conclusions

According to the estimated dietary exposure of Finnish adults to heavy metals, the highest risk is from inorganic arsenic and lead exposures. Cadmium exposure is mainly below the tolerable weekly intake (TWI) determined for kidney damage, but for a part of the adult population the cadmium exposure is at a level where reference [16] found increased osteoporotic fractures. Previously [24], we had assessed that approximately 90% of Finnish children exceed the TWI of cadmium. In the light of these findings, Finland’s derogation to limit the cadmium content of fertilizers, thus lowering its occurrence in plant-based foodstuffs, is appropriate.

The dietary exposure estimates with mainly national data are lower than estimated by EFSA, which was to be expected as the national concentrations for the different foodstuffs are sometimes lower than the European averages. The lead levels in Finnish tap water, in particular, are clearly lower (average <0.1 µg/kg) than the average concentration used by EFSA, 6 µg/kg [19]. Even so, with the current risk management practices, the burden of disease from exposure to inorganic arsenic and to lead means that some cases of cancer and a decrease in the intelligence quotient can be linked to these heavy metals.

The main sources of exposure to inorganic arsenic and lead are cereals (wheat, oats, barley, rye, rice), non-alcoholic drinks and for inorganic arsenic also fish. The importance of dairy as source of lead exposure has decreased as the available monitoring data have included no milk samples with measurable lead content since 2010. For cadmium, roughly one third of the dietary exposure of the average consumer is from cereals. Currently, concerning these exposure sources, the EU legislation (EC No 1881/2006) limits the lead and cadmium content of cereals and arsenic content of rice. For some of the non-alcoholic drinks as well as inorganic arsenic content of different fish species, national data are needed in particular to assess if national risk management actions would be appropriate to decrease the dietary exposure of Finns.

When comparing the estimates of dietary exposure calculated with the same occurrence data but with consumption data from two different years, the exposure for 2012 was slightly higher than for 2007. However, for the most highly exposed group, there was statistical difference only in exposure to cadmium. The trends of heavy metal exposure should be followed in the future, as changes in the typical diet may increase or decrease the dietary exposure of the population as well as the burden of disease from too high an exposure. For example, increased consumption of plant-based foodstuffs may increase the exposure to some of the heavy metals, depending on how the rest of the diet changes, and consequently risk mitigation actions based on risk assessment may be needed. In addition, more information is needed on the typical occurrence of heavy metals in novel plant-based alternatives to meat or dairy products to estimate the future trends more accurately.

## Figures and Tables

**Table 2:** Heavy metal exposure of European adult consumers at middle bound estimate (values below limit of quantification calculated as 50 % of limit of quantification). Exposure is shown as µg/kg bw/d. P95 is the 95th percentile of exposure.

Population Group and Estimate Level	Cd	Pb	iAs	iHg	Ni	Ref.
EU adult population (median of national mean values)	0.25	0.50	0.23	0.059	3.1 ^1^	[13,14,19,20,21]
Finnish adults, mean ^2^	0.22	0.54	0.20	0.051	2.7 ^1^	[13,14,19,20,21]
Finnish 65–74y, mean ^2^	0.20	0.49	0.16	0.050	2.3 ^1^	[13,14,19,20,21]
Finnish adults, P95 ^2^	0.37	0.92	0.32	0.12	5.0 ^1^	[13,14,19,20,21]
Finnish 65–74y, P95 ^2^	0.37	0.78	0.28	0.12	4.0 ^1^	[13,14,19,20,21]

^1^ Value is average of reported lower bound and upper bound estimates. ^2^ Based on FinDiet 2007 consumption data.

**Table 3:** Mean, median (P50) and 95th-percentile (P95) exposures in the different groups, shown as µg/kg bw/d. Results for Cd and Hg should thus be compared with 1/7 of the TWI values shown in Table 2. The 2012 exposure assessment on lead used a dataset where the milk results were limited to the years 2010–2016, all below the limit of reporting, while the 2007 assessment also included older milk data with numerical results. Therefore, the lead results are not directly comparable between the years.

Compound	Population Group, Year	Mean (CI 95%), µg/kg bw/d	P50 (CI 95%), µg/kg bw/d	P95 (CI 95%), µg/kg bw/d
Cd	2007, 25–64Y	0.14 (0.14–0.15)	0.13 (0.13–0.14)	0.24 (0.22–0.27)
	2007, 65–74Y	0.12 (0.12–0.13)	0.12 (0.11–0.12)	0.21 (0.20–0.23)
	2012, 25–64Y	0.16 (0.15–0.17	0.15 (0.14–0.16)	0.27 (0.24–0.29)
	2012, 65–74Y	0.13 (0.12–0.14)	0.12 (0.12–0.13)	0.23 (0.21–0.26)
	2007, 25–45Y men	0.15 (0.14–0.16)	0.14 (0.13–0.15)	0.24 (0.21–0.26)
	2012, 25–45Y men	0.16 (0.15–0.18)	0.15 (0.14–0.17)	0.26 (0.23–0.29)
	2007, 25–45Y women	0.16 (0.15–0.17)	0.15 (0.14–0.16)	0.27 (0.24–0.29)
	2012, 25–45Y women	0.17 (0.16–0.18)	0.16 (0.15–0.17)	0.29 (0.26–0.33)
Pb	2007, 25–64Y	0.19 (0.17–0.21)	0.18 (0.16–0.19)	0.32 (0.29–0.36)
	2007, 65–74Y	0.16 (0.15–0.18)	0.15 (0.14–0.16)	0.28 (0.25–0.31)
	2012, 25–64Y	0.17 (0.15–0.18)	0.15 (0.13–0.16)	0.31 (0.28–0.36)
	2012, 65–74Y	0.13 (0.11–0.14)	0.12 (0.10–0.13)	0.23 (0.19–0.27)
	2007, 25–45Y men	0.21 (0.19–0.24)	0.20 (0.18–0.23)	0.32 (0.28–0.38)
	2012, 25–45Y men	0.17 (0.15–0.19)	0.16 (0.14–0.17)	0.31 (0.27–0.37)
	2007, 25–45Y women	0.22 (0.20–0.24)	0.20 (0.18–0.22)	0.38 (0.33–0.45)
	2012, 25–45Y women	0.19 (0.17–0.22)	0.17 (0.15–0.20)	0.38 (0.33–0.46)
iAs	2007, 25–64Y	0.17 (0.16–0.18)	0.16 (0.15–0.17)	0.28 (0.26–0.30)
	2007, 65–74Y	0.13 (0.12–0.14)	0.12 (0.11–0.13)	0.22 (0.20–0.24)
	2012, 25–64Y	0.18 (0.17–0.19)	0.17 (0.15–0.17)	0.3 (0.28–0.33)
	2012, 65–74Y	0.14 (0.13–0.15)	0.13 (0.12–0.14)	0.24 (0.21–0.26)
	2007, 25–45Y men	0.17 (0.16–0.19)	0.17 (0.16–0.18)	0.28 (0.25–0.30)
	2012, 25–45Y men	0.18 (0.16–0.20)	0.17 (0.16–0.18)	0.31 (0.25–0.37)
	2007, 25–45Y women	0.18 (0.16–0.19)	0.17 (0.15–0.18)	0.29 (0.27–0.32)
	2012, 25–45Y women	0.19 (0.18–0.21)	0.18 (0.17–0.20)	0.33 (0.29–0.37)
iHg	2007, 25–64Y	0.03 (0.03–0.04)	0.03 (0.03–0.03)	0.07 (0.06–0.07)
	2007, 65–74Y	0.03 (0.03–0.04)	0.03 (0.02–0.03)	0.06 (0.05–0.07)
	2012, 25–64Y	0.03 (0.03–0.04)	0.03 (0.03–0.03)	0.07 (0.06–0.07)
	2012, 65–74Y	0.03 (0.03–0.04)	0.03 (0.02–0.03)	0.07 (0.06–0.08)
	2007, 25–45Y men	0.03 (0.03–0.04)	0.03 (0.03–0.04)	0.06 (0.06–0.07)
	2012, 25–45Y men	0.04 (0.03–0.04)	0.03 (0.03–0.04)	0.07 (0.06–0.08)
	2007, 25–45Y women	0.03 (0.03–0.04)	0.03 (0.03–0.03)	0.07 (0.06–0.07)
	2012, 25–45Y women	0.03 (0.03–0.04)	0.03 (0.03–0.04)	0.06 (0.05–0.08)
MeHg	2007, 25–64Y	0.03 (0.03–0.03)	0.02 (0.01–0.02)	0.1 (0.07–0.12)
	2007, 65–74Y	0.05 (0.04–0.06)	0.02 (0.02–0.03)	0.17 (0.13–0.22)
	2012, 25–64Y	0.03 (0.03–0.04)	0.02 (0.01–0.02)	0.11 (0.09–0.13)
	2012, 65–74Y	0.04 (0.03–0.06)	0.02 (0.02–0.03)	0.16 (0.11–0.21)
	2007, 25–45Y men	0.02 (0.02–0.03)	0.02 (0.01–0.02)	0.05 (0.02–0.07)
	2012, 25–45Y men	0.03 (0.02–0.04)	0.01 (0.01–0.02)	0.11 (0.07–0.14)
	2007, 25–45Y women	0.03 (0.02–0.03)	0.02 (0.01–0.02)	0.08 (0.06–0.11)
	2012, 25–45Y women	0.02 (0.02–0.03)	0.02 (0.01–0.02)	0.05 (0.03–0.07)
Ni	2007, 25–64Y	2.43 (2.21–2.71)	2.24 (2.05–2.53)	4.37 (3.93–4.87)
	2007, 65–74Y	2.09 (1.87–2.38)	1.92 (1.72–2.19)	3.8 (3.31–4.37)
	2012, 25–64Y	2.53 (2.31–2.77)	2.31 (2.11–2.54)	4.65 (4.16–5.19)
	2012, 65–74Y	2.16 (1.93–2.39)	1.98 (1.76–2.17)	3.95 (3.38–4.55)
	2007, 25–45Y men	2.31 (2.10–2.63)	2.17 (1.96–2.45)	3.89 (3.49–4.53)
	2012, 25–45Y men	2.40 (2.18–2.72)	2.23 (2.06–2.51)	4.14 (3.40–5.05)
	2007, 25–45Y women	2.79 (2.52–3.12)	2.53 (2.29–2.82)	5.24 (4.58–5.94)
	2012, 25–45Y women	2.89 (2.54–3.23)	2.7 (2.34–2.92)	5.37 (4.57–6.34)

**Table 4:** Risk from dietary exposure of adult (25 to 64 years) and elderly (65 to 74 years) Finns, assessed with the FinDiet 2012 survey data on food consumption. MOE is margin of exposure, determined as health-based guidance value divided by exposure. TWI and TDI are tolerable weekly intake and tolerable daily intake, respectively; BMDL is the lower limit of benchmark dose and the subscript shows the % of change from the baseline. Of the compounds, MeHg is methyl mercury, iHg inorganic mercury and iAs inorganic arsenic.

Compound	Reference Value	Comment on Risk with Estimated Exposure
Cd	TWI 2.5 µg/kg bw/week	0.7% of adults and 0.2% of elderly exceed the TWI. Risk of kidney damage from Cd exposure is thus negligible for more than 99% of the studied population.
	baseline 0.18 µg/kg bw/d	Of women aged 45 to 74 years, 21.5% exceeded this reference level for increased bone breaks and 6% had Cd exposure exceeding level where odds ratio for bone fractures reported in [16] was 3–4 times higher than in group with exposure below the reference level.
Pb	BMDL_01_ 0.50 µg/kg bw/d	At P95 exposure of adults, MOE was 1.5. EFSA considers MOE 10 or higher indicative of negligible risk. Only 2.5% of adults had Pb exposure with MOE 10 or higher.
	BMDL_10_ 0.63 µg/kg bw/d	At P95 exposure of adults, MOE was 1.8. EFSA considers MOE 10 or higher indicative of negligible risk. Only 4.8% of adults had Pb exposure with MOE 10 or higher.
	BMDL_10_ 1.50 µg/kg bw/d	At P95 exposure of adults, MOE was 4.4. EFSA considers MOE 10 or higher indicative of negligible risk. 55% of adults had Pb exposure with MOE 10 or higher.
iAs	BMDL_0.5_ 3.0 µg/kg bw/d	At P95 exposure of adults, MOE was 14.8. Cancer risk in the adult population is low to moderate.
MeHg	TWI 1.3 µg/kg bw/week	1.5% of adults and 3.3% of elderly exceeded the TWI. None of the women aged 25 to 45 years exceeded the TWI. Risk of developmental neurotoxicity through the placenta is therefore negligible.
iHg	TWI 4.0 µg/kg bw/week	None exceeded the TWI, and risk of kidney damage from mercury exposure is therefore negligible.
Ni	TDI 13.0 µg/kg bw/d	0.6% of adults and 0.2% of elderly exceeded the TDI; thus for more than 99% of the population the risk from chronic exposure is negligible. Sensitive people especially in the upper part of the exposure curve may have an acute reaction (dermatitis) from dietary nickel.

**Table 5:** Statistical comparison of mean exposure (in µg/kg bw/d) in three population groups: women (F) and men (M) of 25 to 45 years, and women of 46 to 64 years. The mean exposure was assessed with FinDiet 2012 consumption data. An independent samples *t*-test was used, and equal variances were not assumed.

Compound	25–45Y F	46–64Y F	25–45Y M	*p* (25–45Y F vs. 46–64Y F	*p* (25–45Y F vs. 25–45Y M)
Cd	0.168	0.147	0.162	<0.001	0.258
Pb	0.194	0.153	0.173	<0.001	0.018
iAs	0.197	0.174	0.196	0.045	0.925
iHg	0.033	0.033	0.035	0.709	0.159
MeHg	0.020	0.039	0.026	0.001	0.179
Ni	2.872	2.565	2.456	0.008	0.001

**Table 6:** Statistical comparison of mean exposure (in µg/kg bw/d) of lowest and highest weight quartile among the whole population group studied in FinDiet 2012 (25 to 74 years, both genders). Independent samples *t*-test was used, and equal variances were not assumed.

Compound	Weight Quartile 1 (up to 65.9 kg)	Weight Quartile 4 (above 87.3 kg)	*p*
Cd	0.178	0.117	<0.001
Pb	0.187	0.124	<0.001
iAs	0.216	0.140	<0.001
iHg	0.039	0.027	<0.001
MeHg	0.035	0.029	0.272
Ni	3.246	1.819	<0.001

**Table 7:** Statistical comparison of the average exposure (in µg/kg bw/d) in the age group 25 to 45 years, compared between the two years. In the first columns, the age groups were not divided by gender, while the right-hand columns only compare women of 25 to 45 years. Individual daily exposure values were used, and in the *t*-test, equal variances were not assumed.

	Both Genders, 25–45Y			Only Women, 25–45Y		
**Compound**	**2007**	**2012**	***p***	**2007**	**2012**	***p***
Cd	0.15	0.17	<0.001	0.16	0.17	0.007
Pb	0.18	0.18	0.582	0.20	0.19	0.907
iAs	0.18	0.20	0.045	0.18	0.20	0.126
iHg	0.03	0.03	0.912	0.03	0.03	0.711
MeHg	0.02	0.02	0.743	0.03	0.02	0.144
Ni	2.60	2.69	0.219	2.83	2.87	0.711

## Data Availability

The occurrence data used for exposure assessment are available as food subgroup level summaries in Appendix 2 of ref. [25]. The individual food consumption data are available according to the data management policies and procedures of the Finnish Institute of Health and Welfare THL.

## Supplementary Materials

The following are available online at https://www.mdpi.com/article/10.3390/ijerph182010581/s1, Table S1: Sources of cadmium exposure for the average consumer in different age groups and at different years; Table S2: Sources of lead exposure for the average consumer in different age groups and at different years; Table S3: Sources of inorganic arsenic exposure for the average consumer in different age groups and at different years; Table S4: Sources of inorganic mercury exposure for the average consumer in different age groups and at different years; Table S5: Sources of nickel exposure for the average consumer in different age groups and at different years; Table S6. Sources of methyl mercury exposure for the average consumer in different age groups and at different years; Table S7: Sources of exposure for the high consumer (95th percentile) in the age group 25 to 64 years, according to consumption data from FinDiet 2012; Table S8. Mean dietary exposure (µg/kg bw/d) according to middle bound estimate in different age groups of FinDiet 2007 and FinDiet 2012 surveys, separated by gender; Table S9: Limits of quantification (LOQ), in mg/kg, of the analysis methods used for the occurrence data in Finland.
