# Supplementary material for: Dietary Heavy Metal Exposure among Finnish Adults in 2007 and in 2012
Source: Int J Environ Res Public Health. 2021 Oct 9;18(20):10581. doi: 10.3390/ijerph182010581 (PMC8535204; doi:10.3390/ijerph182010581)
Supplement: Supplementary file 1 [file ijerph-18-10581-s001.zip › ijerph-1384851-supplementary.pdf]

International Journal of
Environmental Research and Public Health

MDPI

Article

Dietary Heavy Metal Exposure among Finnish Adults in 2007 and in 2012

Johanna Suomi [1,*], Liisa Valsta [2] and Pirkko Tuominen [1]

[1] Risk Assessment Unit, Finnish Food Authority, 00790 Helsinki, Finland; pirkko.tuominen@ruokavirasto.fi
[2] Population Health Unit, Finnish Institute for Health and Welfare, 00271 Helsinki, Finland; liisa.valsta@thl.fi
* Correspondence: johanna.suomi@ruokavirasto.fi or alias johanna.suomi@foodauthority.fi

Citation: Suomi, J.; Valsta, L.; Tuominen, P. Dietary Heavy Metal Exposure among Finnish Adults in 2007 and in 2012. *Int. J. Environ. Res. Public Health* **2021**, *18*, 10581. https://doi.org/10.3390/ijerph182010581

Academic Editor: Martin David Rose

Received: 1 September 2021
Accepted: 7 October 2021
Published: 9 October 2021

Publisher's Note: MDPI stays neutral with regard to jurisdictional claims in published maps and institutional affiliations.

Copyright: © 2021 by the authors. Licensee MDPI, Basel, Switzerland. This article is an open access article distributed under the terms and conditions of the Creative Commons Attribution (CC BY) license (https://creativecommons.org/licenses/by/4.0/).

Supplementary Materials

Table S1. Sources of cadmium exposure (% of total exposure shown in Table 3) for the average consumer in different age groups and at different years. Consumption data used in the assessment were from the surveys FinDiet 2007 and FinDiet 2012. Results for men and women in the 25- to 45-year (25–45Y) age group in 2012 are presented separately; they are also included in the group 25–64Y for 2012. Middle bound scenario was used for non-detects.

Food group	2007<br>25–64Y	2007<br>65–74Y	2012<br>25–64Y	2012<br>65–74Y	2012<br>25–45Y men	2012<br>25–45Y women
Cereals	32.2	36.1	31.0	33.9	31.6	30.1
Vegetables	14.4	14.8	15.6	17.6	13.9	15.9
Starchy roots	12.5	13.8	9.1	10.9	10.2	8.4
Legumes, nuts, oilseeds	3.3	3.4	3.9	2.6	3.1	4.4
Fruit, berries	5.0	6.8	4.5	6.2	2.9	4.8
Meat and offal	4.2	5.0	4.2	4.0	3.3	3.1
Fish and seafood	2.1	2.0	1.9	4.0	1.6	1.9
Dairy	6.2	5.5	6.1	5.3	6.6	6.7
Eggs	0.7	0.8	0.8	0.8	1.0	0.8
Sugar and sweets	8.0	3.3	6.5	3.4	6.3	8.5
Fats and oils	0.2	0.1	0.2	0.1	0.3	0.2
Juices	5.4	3.7	4.6	3.4	5.6	5.0
Non-alcoholic drinks	1.6	1.4	1.6	1.3	1.3	2.2
Alcohol	0.8	0.4	0.7	0.4	0.8	0.5
Drinking water	0.6	0.6	0.6	0.4	0.4	0.6
Spices	1.5	1.1	1.6	1.0	2.3	1.5
Weight loss products	0.0	0.0	0.3	0.0	0.3	0.4
Combination foods	0.0	0.0	6.6	4.4	8.3	4.8
Supplements	1.3	1.3	0.3	0.2	0.4	0.2

Table S2. Sources of lead exposure (% of total exposure shown in Table 3) for the average consumer in different age groups and at different years. Consumption data used in the assessment were from the surveys FinDiet 2007 and FinDiet 2012. Results for men and women in the 25- to 45-year (25–45Y) age group in 2012 are presented separately; they are also included in the group 25–64Y for 2012. Middle bound scenario was used for non-detects. The concentration data for dairy products were different between the years as no milk samples were above the limit of detection after 2010. That is why the relative importance of dairy is so much lower in 2012.

Food group	2007 25–64Y	2007 65–74Y	2012 25–64Y	2012 65–74Y	2012 25–45Y men	2012 25–45Y women
**Cereals**	17.8	20.4	15.4	18.2	16.7	15.1
**Vegetables**	6.1	6.6	9.1	9.8	8.8	9.0
**Starchy roots**	3.7	4.3	3.6	4.9	3.9	3.0
**Legumes, nuts, oilseeds**	1.0	0.8	2.8	2.5	2.4	2.9
**Fruit, berries**	6.8	9.1	8.3	14.1	4.3	7.3
**Meat and offal**	4.6	5.1	4.8	5.2	5.2	3.2
**Fish and seafood**	1.5	1.9	1.9	3.9	1.6	1.6
**Dairy**	17.3	18.7	4.6	3.7	4.3	4.9
**Eggs**	0.0	0.0	0.0	0.0	0.0	0.0
**Sugar and sweets**	7.8	3.4	7.8	4.5	8.6	9.9
**Fats and oils**	0.3	0.5	0.4	0.6	0.5	0.3
**Juices**	5.4	4.0	7.5	5.9	9.2	7.8
**Non-alcoholic drinks**	11.8	11.0	12.5	10.9	10.5	14.3
**Alcohol**	5.3	3.5	5.4	5.3	5.9	3.0
**Drinking water**	2.1	2.1	2.6	2.3	2.1	2.5
**Spices**	5.4	5.9	5.8	6.3	7.2	4.8
**Weight loss products**	0.3	0.1	6.4	0.7	7.4	9.4
**Combination foods**	0.0	0.0	0.4	0.3	0.5	0.3
**Supplements**	2.7	2.7	0.8	0.6	1.0	0.6

Table S3. Sources of inorganic arsenic exposure (% of total exposure shown in Table 3) for the average consumer in different age groups and at different years. Consumption data used in the assessment were from the surveys FinDiet 2007 and FinDiet 2012. Results for men and women in the 25- to 45-year (25–45Y) age group in 2012 are presented separately; they are also included in the group 25–64Y for 2012. Middle bound scenario was used for non-detects.

Food group	2007 25–64Y	2007 65–74Y	2012 25–64Y	2012 65–74Y	2012 25–45Y men	2012 25–45Y women
Cereals	14.4	18.8	14.1	16.8	13.8	14.9
Vegetables	7.9	5.6	7.8	8.6	6.9	8.6
Starchy roots	0.0	0.0	0.0	0.0	0.0	0.0
Legumes, nuts, oilseeds	0.5	0.3	0.9	0.6	0.8	1.0
Fruit, berries	3.4	4.5	3.2	4.5	2.4	3.4
Meat and offal	3.1	3.9	3.0	3.3	3.3	2.4
Fish and seafood	11.3	7.5	13.5	12.0	16.6	13.2
Dairy	6.9	5.7	7.0	5.5	6.8	8.2
Eggs	0.2	0.2	0.2	0.2	0.2	0.2
Sugar and sweets	4.6	3.6	4.1	3.0	4.1	5.2
Fats and oils	2.7	3.6	2.7	3.2	2.3	2.6
Juices	8.1	6.5	6.9	5.6	8.2	7.6
Non-alcoholic drinks	22.6	25.4	22.3	24.8	19.3	20.7
Alcohol	4.3	1.9	3.5	2.6	4.4	2.0
Drinking water	3.8	4.4	3.9	3.3	3.0	4.0
Spices	4.0	5.5	4.6	4.4	5.0	3.8
Weight loss products	0.0	0.0	0.4	0.0	0.4	0.6
Combination foods	0.0	0.0	1.5	1.1	1.8	1.1
Supplements	2.2	2.6	0.7	0.4	0.6	0.4

Table S4. Sources of inorganic mercury exposure (% of total exposure shown in Table 3) for the average consumer in different age groups and at different years. Consumption data used in the assessment were from the surveys FinDiet 2007 and FinDiet 2012. Results for men and women in the 25- to 45-year (25–45Y) age group in 2012 are presented separately; they are also included in the group 25–64Y for 2012. Middle bound scenario was used for non-detects.

Food group	2007 25–64Y	2007 65–74Y	2012 25–64Y	2012 65–74Y	2012 25–45Y men	2012 25–45Y women
Cereals	0.8	0.3	1.1	0.7	1.4	1.3
Vegetables	13.5	12.2	16.0	16.4	14.8	18.0
Starchy roots	5.8	6.8	4.8	6.0	5.1	4.5
Legumes, nuts, oilseeds	1.0	0.9	1.4	1.0	1.3	1.6
Fruit, berries	5.8	6.9	5.9	7.5	3.8	6.7
Meat and offal	10.6	11.5	11.0	11.3	12.2	8.2
Fish and seafood	20.5	30.6	21.6	28.2	17.3	14.8
Dairy	0.3	0.2	0.3	0.2	0.2	0.4
Eggs	0.0	0.0	0.0	0.0	0.0	0.0
Sugar and sweets	6.4	5.6	7.0	5.9	6.0	8.9
Fats and oils	1.4	1.4	1.5	1.5	1.5	1.6
Juices	18.9	12.2	18.6	13.1	22.9	23.0
Non-alcoholic drinks	2.0	0.5	2.2	1.1	3.4	3.0
Alcohol	3.0	1.0	2.6	1.7	3.5	1.5
Drinking water	0.8	0.8	0.8	0.7	0.7	1.2
Spices	5.4	5.8	4.1	4.0	4.3	4.2
Weight loss products	0.0	0.0	0.1	0.0	0.1	0.2
Combination foods	0.0	0.0	0.2	0.1	0.3	0.2
Supplements	3.7	3.4	0.8	0.6	1.1	0.8

Table S5. Sources of nickel exposure (% of total exposure shown in Table 3) for the average consumer in different age groups and at different years. Consumption data used in the assessment were from the surveys FinDiet 2007 and FinDiet 2012. Results for men and women in the 25- to 45-year (25–45Y) age group in 2012 are presented separately; they are also included in the group 25–64Y for 2012. Middle bound scenario was used for non-detects.

Food group	2007 25–64Y	2007 65–74Y	2012 25–64Y	2012 65–74Y	2012 25–45Y men	2012 25–45Y women
Cereals	22.6	32.9	20.1	25.5	21.1	18.8
Vegetables	7.2	7.6	8.1	9.4	7.1	7.7
Starchy roots	4.7	5.8	3.7	4.8	4.3	3.1
Legumes, nuts, oilseeds	8.4	6.3	15.3	11.5	13.9	15.3
Fruit, berries	9.4	11.3	9.4	12.4	6.2	9.5
Meat and offal	3.1	2.9	3.1	2.9	4.0	2.8
Fish and seafood	0.4	0.6	0.4	0.7	0.4	0.3
Dairy	8.3	8.3	8.5	8.1	9.0	9.2
Eggs	0.2	0.2	0.3	0.3	0.3	0.3
Sugar and sweets	16.1	5.8	12.1	5.6	12.7	15.0
Fats and oils	6.2	7.4	6.1	7.1	5.9	5.7
Juices	4.2	3.0	3.1	2.5	4.0	3.1
Non-alcoholic drinks	6.2	5.4	6.3	6.5	6.6	6.2
Alcohol	0.7	0.5	0.6	0.6	0.8	0.3
Drinking water	0.7	0.7	0.7	0.6	0.6	0.7
Spices	1.1	1.1	1.6	1.2	2.2	1.3
Weight loss products	0.0	0.0	0.2	0.0	0.3	0.3
Combination foods	0.0	0.0	0.4	0.2	0.5	0.3
Supplements	0.3	0.3	0.1	0.1	0.1	0.1

Table S6. Sources of methyl mercury exposure (% of total exposure shown in Table 3) for the average consumer in different age groups and at different years. Consumption data used in the assessment were from the surveys FinDiet 2007 and FinDiet 2012. Results for men and women in the 25- to 45-year (25–45Y) age group in 2012 are presented separately; they are also included in the group 25–64Y for 2012. Middle bound scenario was used for non-detects. As methyl mercury was assumed to be present only in fish and seafood, results are shown at higher detail than in Tables S1–S5.

Species	2007 25–64Y	2007 65–74Y	2012 25–64Y	2012 65–74Y	2012 25–45Y men	2012 25–45Y women
**Perch**	1.7	8.1	5.8	4.2	2.4	0.0
**Pike**	15.7	17.3	14.5	5.3	8.9	0.0
**Rainbow trout**	13.0	10.8	13.7	13.8	16.7	23.1
**Salmon**	3.2	3.2	6.8	5.8	5.8	10.4
**Average fish (***	37.3	44.7	28.6	50.9	28.3	15.8
**Vendace**	3.5	5.5	7.7	4.8	9.4	2.9
**Saithe**	4.9	2.5	4.7	2.4	6.6	8.6
**Tuna**	14.1	1.9	9.8	6.1	13.0	27.3
**Other fish**	4.1	5.7	6.1	5.4	4.9	7.3
**Roe**	0.1	0.1	0.2	0.2	0.1	0.2
**Molluscs**	0.2	0.0	0.1	0.3	0.1	0.4
**Crustaceans**	2.0	0.3	2.0	0.8	3.8	4.0

(* Not identified by species in the consumption data, calculated as mixture of perch, pike, vendace and Baltic herring in the nutritional assessments. In the current study, a mixture of 1:1:1:1 was assumed.

Table S7. Sources of exposure for the high consumer (95th percentile) in the age group 25 to 64 years, according to consumption data from FinDiet 2012. Middle bound scenario was used for non-detects. At this food grouping level, methyl mercury would be 100% from fish and seafood.

Food group	Cd	Pb	inorg. As	inorg. Hg	Ni
**Cereals**	13.4	4.0	4.3	0.2	6.3
**Vegetables**	6.8	3.5	17.1	10.9	2.7
**Starchy roots**	3.5	0.8	0.0	1.6	0.8
**Legumes, nuts, oilseeds**	9.5	1.9	0.2	0.4	62.9
**Fruit, berries**	2.2	2.6	1.0	2.4	4.6
**Meat and offal**	12.1	1.1	0.4	7.9	0.7
**Fish and seafood**	0.9	0.4	60.2	53.2	0.1
**Dairy**	2.6	7.3	1.9	0.0	2.4
**Eggs**	0.3	0.0	0.0	0.0	0.1
**Sugar and sweets**	6.8	5.0	0.8	1.4	12.1
**Fats and oils**	0.1	0.1	0.5	0.5	1.3
**Juices**	2.9	3.8	1.5	17.8	0.9
**Non-alcoholic drinks**	0.8	11.9	3.5	0.9	4.0
**Alcohol**	0.3	2.5	1.1	0.7	0.1
**Drinking water**	0.2	1.1	0.7	0.2	0.2
**Spices**	0.6	1.5	4.9	1.4	0.2
**Weight loss products**	0.1	50.7	0.1	0.0	0.3
**Combination foods**	36.9	0.2	0.3	0.1	0.3
**Supplements**	0.1	1.4	1.5	0.3	0.0

Table S8. Mean dietary exposure (µg/kg bw/d) according to middle bound estimate in different age groups of FinDiet 2007 and FinDiet 2012 surveys, separated by gender. For the FinDiet 2012 survey, lead exposure was estimated with milk occurrence data from 2006 onwards, while the exposure for the older survey was estimated with some older numerical data included. For comparison between the years, the indicative lead exposure estimated with the milk occurrence data of 2006 onwards is also shown for the 2007 consumption.

Year	Gender	Age group	Cd	Pb (old data)	Pb	iAs	iHg	MeHg	Ni
**2007**	FEMALE	25–34	0.164	0.256	0.224	0.185	0.034	0.023	2.939
	MALE		0.155	0.210	0.172	0.186	0.035	0.014	2.421
**2012**	FEMALE		0.183	-	0.204	0.214	0.036	0.024	3.169
	MALE		0.176	-	0.190	0.223	0.038	0.029	2.507
**2007**	FEMALE	35–44	0.151	0.200	0.175	0.175	0.033	0.029	2.756
	MALE		0.143	0.186	0.158	0.177	0.035	0.028	2.241
**2012**	FEMALE		0.154	-	0.189	0.186	0.030	0.017	2.544
	MALE		0.151	-	0.163	0.177	0.034	0.026	2.425
**2007**	FEMALE	45–54	0.141	0.182	0.162	0.171	0.034	0.038	2.582
	MALE		0.135	0.194	0.164	0.172	0.032	0.027	2.243
**2012**	FEMALE		0.160	-	0.161	0.195	0.035	0.035	2.655
	MALE		0.145	-	0.144	0.175	0.039	0.050	2.173
**2007**	FEMALE	55–64	0.134	0.168	0.148	0.162	0.032	0.033	2.412
	MALE		0.125	0.163	0.137	0.168	0.034	0.045	2.066
**2012**	FEMALE		0.140	-	0.147	0.156	0.031	0.041	2.602
	MALE		0.143	-	0.137	0.164	0.033	0.042	2.492
**2007**	FEMALE	65–74	0.122	0.158	0.132	0.123	0.032	0.052	2.119
	MALE		0.126	0.169	0.142	0.134	0.032	0.042	2.058
**2012**	FEMALE		0.136	-	0.128	0.154	0.030	0.034	2.350
	MALE		0.133	-	0.125	0.138	0.034	0.050	2.025

Table S9. Limits of quantification (LOQ), in mg/kg, of the analysis methods used for the occurrence data in Finland. All of the analyses were performed with validated laboratory methods. If the value is given as "or", the method is validated for more than one analysis equipment of same description but with different LOQ, or the data are from several laboratories. Dash (-) signifies the analysis method had not been used for this heavy metal in the occurrence dataset used in the article.

Analysis method	Matrix	Cd	Pb	As	Hg	Ni	Al
**AAS**	Foodstuffs of animal origin	0.001	0.01	-	0.001	0.02	-
**AAS (graphite oven)**	Foodstuffs of animal origin or plant-based foods	-	-	-	0.05 or 0.005	-	-
**ICP-MS**	Foodstuffs of animal origin	0.01 or 0.001	0.01	0.01 or 0.005 or 0.001	-	0.02	0.20
**ICP-MS**	Plant-based foodstuffs	0.01 or 0.001	0.05 or 0.02 or 0.005	0.01	-	0.02	2.0
**ICP-MS**	Cereal products	0.01 or 0.005 or 0.001	0.05 or 0.02 or 0.005	0.02	-	0.10	5.0 (pasta)
**HPLC-ICP-MS**	Rice	-	-	0.04 (iAs)	-	-	-
